# Detonation Velocity Measurements Using Rare-Earth Doped Fibres

**DOI:** 10.3390/s19071697

**Published:** 2019-04-10

**Authors:** Josh Pooley, Ed Price, James W. Ferguson, Morten Ibsen

**Affiliations:** 1 Optoelectronics Research Centre, University of Southampton, Southampton SO17 1BJ, UK; j.pooley@soton.ac.uk; 2 AWE Aldermaston, Reading, Berkshire RG7 4PR, UK; ed.price@awe.co.uk (E.P.); james.ferguson@awe.co.uk (J.W.F.)

**Keywords:** fibre sensing, rare-earth fibres, detonation, velocity sensing

## Abstract

In this paper, a simple detonation velocity measurement scheme is presented, which exploits the length-dependent amplified spontaneous emission (ASE) power emitted by off-the-shelf Er-doped fibres. This measurement scheme is first calibrated using cutback tests, so that minimal processing is required between data collection and velocity readout. We then demonstrate the use of this method in an explosive cylinder test and achieve a spatial resolution of approximately ±2 mm, owing to its implementation in a helical geometry. Alongside the standard Er fibres, a specially made, high-concentration Er/Yb-doped fibre is also calibrated, which demonstrates a potential spatial resolution approaching ±20 μm.

## 1. Introduction

An important parameter when characterising the performance and behaviour of an explosive compound is the detonation velocity [1]. Detonation velocity is defined as the rate at which a chemically sustained shockwave propagates through an explosive medium [2].

Over several decades, a number of detonation velocity measurement techniques have been developed, such as the high-speed photographic streak camera method [2]. This technique is capable of nanosecond scale temporal resolutions but is often limited to a ∼cm scale measurement range and requires a direct sight line between the camera and the subject.

Many simple and more versatile ways of measuring detonation velocity work on a Time-of-Arrival (TOA) basis, where probes are in direct contact with an explosive charge. When a part of the TOA probe is destroyed by the propagating shock front, a disturbance is measured and recorded on a data acquisition device (DAQ). This puts a time stamp on the shock front at known spatial intervals and enables the tracking of the detonation velocity throughout the event.

A contemporary example of a discrete TOA device, demonstrated by Shinas and Doty [3], uses aluminium-coated optical fibres as sacrificial probes, where the reflected signal drops as soon as the shock front disturbs the aluminium layer. Discrete TOA measurement techniques, such as this, are reliable and simple as long as the separation of each probe is known to a high precision. However, they must often make a compromise between the spatial resolution and measurement length—in this example, a high resolution, 250 μm probe separation, was setup to measure over an area of 1.25 × 1.25 mm.

Continuous TOA devices aim to extend detonation velocity measurements into the cm–m length scale whilst maintaining a spatial resolution that is a ≤mm scale. A popular continuous TOA device over the last decade has been the chirped fibre Bragg grating (CFBG) [4,5,6,7]. In this case, the detonation velocity is measured by illuminating a linearly chirped fibre Bragg grating (CFBG) with a broadband light, usually from an amplified spontaneous emission (ASE) source, and by inferring the erasure velocity from the monotonically decreasing reflection bandwidth. A typical measurement range, in this case, is around 100 mm and with a spatial resolution of 50 μm–1 mm [8].

Due to the destruction of the probes in each test, some TOA methods can become costly over multiple trials or long distance measurements. In this paper, we demonstrate a simple, scalable, and cheap method for a continuous measurement of the detonation velocity, which utilises Er and Er/Yb-doped optical fibres.

The general principle, in this case, is to exploit the length dependence of the total fluorescent power captured from a pumped active fibre. This concept was first implemented by J.D. Weiss in 1994 [9], who used Neodymium and Phosphorous co-doped, elongated-cladding optical-fibre probes to measure shock propagation in up to 19 m lengths of detonating cord.

In the work presented here, both off-the-shelf Er fibre and specially made, high-concentration Er/Yb fibre is pre-calibrated in cutback tests so that the length–power dependency is linearised and post-processing is minimised. The aim is to demonstrate a cheaper and easier implementation of an active fibre velocity probe.

As a full demonstration of the calibrated device, an off-the-shelf Er fibre probe is used to measure the detonation velocity continuously over a 1 m length and in a helical geometry—a setup that results in a tenfold increase in the spatial resolution, compared to a 0.1 m long, linearly mounted probe.

## 2. Concept

**Figure 1:**
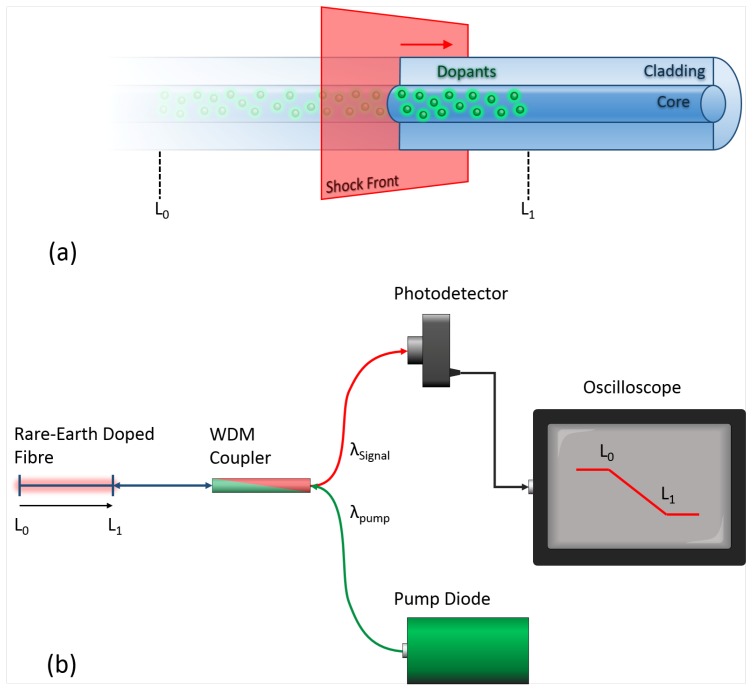
(**a**) An illustration of the active fibre concept—a detonation shock wave destroys the active fibre, reducing the number of fluorescent dopants. (**b**) A schematic showing the basic setup for an active fibre detonation velocity probe. A rare-earth-doped fibre is pumped with a diode laser. The amplified spontaneous emission (ASE) fluorescence is filtered through a wavelength division multiplexing (WDM) coupler and collected on a photodetector.

In the system presented here, an Er- or Er/Yb-doped fibre is embedded in an explosive and optically pumped with a laser diode. This induces spontaneous emission, which is omnidirectional and can be monitored on a photodetector. As the active fibre region is consumed by the detonation front, the rare-earth emission sources are depleted and the power measured by the detector decreases [9]. This concept is illustrated in Figure 1a.

If the detector voltage can be monitored at a sufficient speed, the evolution of the shock front position over time can be recorded. A typical interrogation setup is shown in Figure 1b, where a wavelength division multiplexing (WDM) coupler is used to separate the pump and signal wavelengths.

In order for the fluorescent signal power to scale linearly with length, the pump power absorption at the front end must be balanced with the higher gain (amplified spontaneous emission—ASE) from the back end. In this case, a trial and error approach was taken, with various pump powers and probe lengths tested in laboratory conditions—akin to the damage assessment calibration tests on Er fibre by Fan et al. [10]. These so-called cutback tests are detailed in the next section.

The major benefits of this active fibre system, when compared to CFBG techniques, are cost, usability, and scalability:

- The prevalence of certain rare-earth-doped fibres, particularly in the telecoms industry, means that high volumes of these fibres are manufactured and that costs are low. A common Er-doped fibre may cost in the region of £10–20 per meter.
- For consolidated charges, the inherent sensitivity of CFBGs to strain and temperature nonuniformities can make them challenging to implement. The relative insensitivity of active fibres to these stimuli means that they can be used more liberally and by nonspecialists.
- Due to the structural intricacy of a CFBG, the fabrication of probes exceeding a few tens of centimeters can be difficult and expensive. This is not the case for active fibre probes, which can be scaled to multimetre lengths if necessary, depending on the characteristics of the fibre and pump light.

## 3. Cutback Tests

To test this concept and to calibrate the active fibre probes, a system similar to Figure 1 was assembled, where the photodetector was replaced by a power meter, and the pump power from a 977 nm pump diode was monitored using a 50–50 splitter. As a controlled way to simulate the destructive effects of a detonation front, a razor blade was used to cut small pieces from the end of the active fibre.

Between successive cuts, the fibre length was measured and a drop of index-matching fluid was applied to the cut end. The spontaneous emission was then measured on the power meter, and the process was repeated until the entire active region was destroyed.

**Figure 2:**
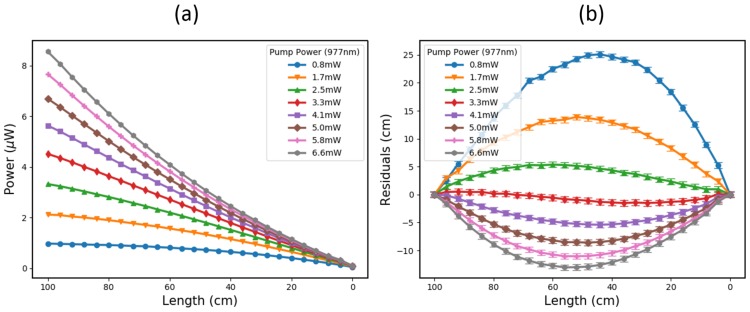
(**a**) Shows the ASE Power vs Length of a 1 m-long Er-doped fibre (Fibrecore I-12) for a range of pump powers. (**b**) Shows the residuals compared to an ideal, linear Power vs Length relationship. The uncertainty bars correspond to the ±0.5 cm cutback precision.

As an initial optimization of this system, cutback tests were performed on three different concentrations of Er- and Er/Yb-doped fibre. Each fibre was also tested at different pump powers to get an optimal linearity. The results are shown in Figure 2 for a one-meter length of Fibrecore I-12 Er fibre, which has an absorption of 11.6 dB/m (for a 980 nm pump wavelength). The length was cut back by 4 cm at a time and measured using a ruler to a precision of around ±0.5 cm.

It can be seen here that when the pump power is too low (≤2.5 mW in this case), the back end of the active fibre is not exposed to sufficient pump light and, therefore, has a reduced contribution to the power reaching the detector. This causes the power to scale nonlinearly with the length in a convex way [10].

On the other hand, when the pump power is high enough to excite the active molecules uniformly along the length (≥4.1 mW here), the power scales nonlinearly in a concave way [10]. This is thought to be due to the accumulated gain that is incurred when back-end emissions stimulate front-end excited states.

An optimally linear correspondence between the length and emitted power is, therefore, achieved through an adjustment of the following five parameters: dopant type, dopant concentration, pump power, pump wavelength, and fibre length.

**Figure 3:**
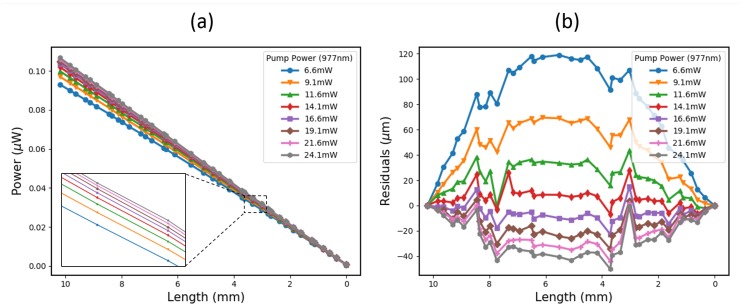
(**a**) Shows the ASE Power vs Length of a 10-mm-long, high-concentration (>1000 dB/m at 980 nm) Er/Yb-doped fibre for a range of pump powers. (**b**) Shows the residuals compared to an ideal, linear Power vs Length relationship. The cutback precision was ±20 μm.

As a demonstration of the effects of the dopant concentration and constituents on the power–length relationship of an active fibre probe, a cutback test was conducted on a 10 mm-long, high-concentration Er/Yb fibre. The results are shown in Figure 3.

To get this many cutback measurements from a 10-mm probe length, a microscope was used that could pick up any visible fluorescence from the pumped fibre. By applying some index-matching fluid to the probe end, its length could be measured using a micrometer. The measurement precision achieved using this technique was around ±20 μm, limited by lensing effects from the fluid, the microscope screen resolution, and the microscope alignment stability.

From Figure 3, it is clear that a much higher pump power is required to excite the active molecules throughout the 10-mm-long probe, because the pump absorption is so extreme—more than 1000 dB/m at 980 nm. This limits the useful length over which the fibre can be used as a detonation velocity probe, but the power per unit length is higher than the lower concentration Er fibre shown in Figure 2.

For a 3.3 mW pump power in the lower concentration I-12 fibre, the total fluorescent power from a 1 m length is ∼4.5 μW. This is equivalent to 4.5 nW/mm. For the high concentration Er/Yb fibre in Figure 3, the power per unit length is over twice as much, at 10 nW/mm.

As a velocity probe, the high-concentration Er/Yb fibre is also expected to be highly linear, with residuals of approximately ±20 μm over a 10-mm length. Whilst this fibre was not carried through to the explosive testing stage, due to the low total signal power, it does show promise as a high-resolution probe for the future, offering potentially competitive spatial uncertainties down into the tens of microns.

A comparison of these fibres, as well as an intermediary Er fibre (Fibrecore I-25) is shown in Table 1. This table shows the approximate length scales over which each fibre type could produce a linear erasure response (maximum residuals less than ∼2% of the total length), as well as the optimal pump powers.

## 4. Explosive Test Setup

**Figure 4:**
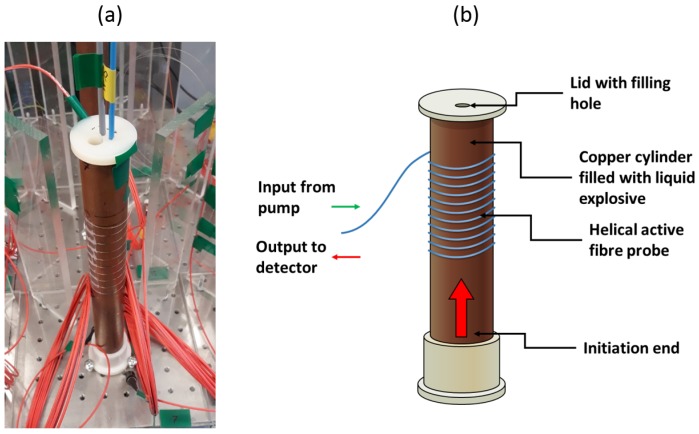
(**a**) A photograph showing the cylinder test setup, with a 1-m length of Er-doped fibre wrapped around in a helix. (**b**) An annotated schematic showing the same cylinder test setup.

In order to demonstrate the active fibres as linearised detonation velocity probes, a test was set up in which active fibres were attached to a copper cylinder filled with a liquid explosive—sensitised nitromethane. The copper cylinder was 30 cm long, with a ∼3.2 cm outside diameter and a ∼0.3 cm wall thickness. To show the scalability of this concept and to improve the spatial resolution of the measurement tenfold, a 1-m length of Fibrecore I-12 Er fibre was wound around the cylinder in a helical fashion (Figure 4).

The back-end setup that was used to pump and interrogate this Er fibre probe is shown in Figure 1. In this case, a 125 MHz New Focus 1811-FC detector was used to monitor the fluorescent signal, with the output voltage monitored on a 1.25 GSamples/Second, 10-bit National Instruments PXIe-5160 DAQ system. To ensure that the operation of the active fibre probe was as linear as possible, the pump power was set to ∼3.2 mW (with a 977 nm pump diode) in conjunction with the calibration shown in Figure 2.

Inside the copper tube, a length of standard telecom’s fibre was mounted in order to record a heterodyne velocimetry (HetV) trace [11]. HetV is an interferometric technique for measuring velocities in free-space or in certain liquid explosive tests [12]. In these tests, HetV is used as a confirmatory measurement alongside the active fibre probes. However, in experiments requiring detonation velocity measurements in consolidated charges, HetV would be unsuitable.

## 5. Results and Discussion

**Figure 5:**
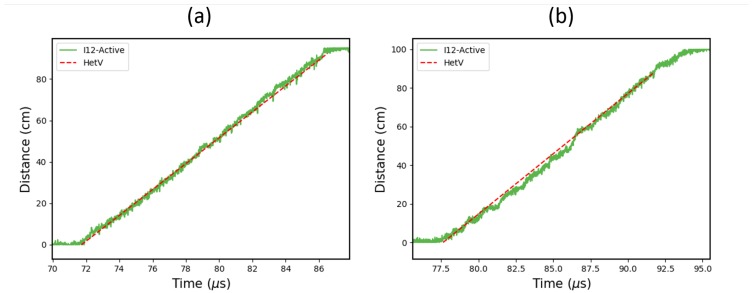
Plots showing the data from two 1-m long Er-doped fibre detonation velocity tests (Fibrecore I-12). For comparison, the data has been overlaid with the corresponding HetV results. The results shown in (**a**,**b**) are from two nominally identical cylinder tests. However, a substructure, thought to be due to the helical mounting geometry, is particularly present in Figure 5b.

The results from the cylinder tests are shown in Figure 5. These plots show the data from the helical Er fibre probes as well as the corresponding HetV data from each test. In order to make a direct comparison between the two data sets, the HetV velocity–time trace has been integrated to yield a distance–time graph.

Since the active fibre probes have been pre-calibrated to operate as linearly as possible, they do not require any significant post-processing—an inevitability in the Bragg grating techniques, where the spectral characteristics of each grating and source can vary. Whilst linearisation is not essential to the operation of the active fibre probe technique, it allows the measured detector signal to be calibrated in a particularly simple way.

In this case, the raw voltage data from the DAQ has been normalised, giving the fraction of the remaining probe length, and then multiplied by the full length of the probe in order to obtain a distance–time plot for the progressing detonation wave. This data has also been low-pass filtered (30 MHz cutoff) to remove any high-frequency noise.

The plots in Figure 5 show that the active fibre probe data agrees well with the HetV record. By taking a linear regression fit of the HetV and active fibre probe data, the average detonation velocity from each measurement can be obtained and compared. The measured average velocities from the HetV probes are 6.25 km/s and 6.24 km/s, which correspond to the average velocities from the active fibres, 6.37 km/s and 6.34 km/s. Taking the HetV measurements as the benchmark, there is a velocity discrepancy of 1.9% and 1.6% for the two active fibre probes, respectively. This is likely to be due to the ±2.5% uncertainty in the probe length, which is a result of the practical difficulty when mounting the fibre in a helical arrangement.

A substructure is particularly visible in Figure 5b which corresponds to the ten full turns of the helical probe. This nonuniformity was anticipated because the helical setup was constructed manually, but it happens to inadvertently demonstrate the ability of the probe to pick up changes in the velocity over 10 cm length scales (for a 1 m long test) or just 1 cm length scales in the shockwave propagation direction.

By taking the standard deviation of the signal noise from these cylinder tests, the spatial uncertainty from this setup can be gleaned. This was done for both unfiltered and 30 MHz low-pass filtered data, yielding percentage uncertainties of approximately ±1.6% and ±0.9%, respectively. Over the 0.1 m axial length of the helix, this corresponds to a maximum spatial uncertainty/resolution of ±1.6 mm and ±0.9 mm. The uncertainty due to the calibration residuals, shown in Figure 2, is in a similar range, at around ±2 mm.

Similar results were obtained in the crack detection tests by Fan et al. [10], who managed a statistical error of just under 3 mm over a 0.4 m length. However, this was achieved using an optical spectrum analyser in place of the fast photodetectors used here and with a higher pump power.

Whilst a similar analysis was not done explicitly for the Nd/P-doped fibres used in Weiss’s work [9], it is our estimate, based on an inspection of the data presented, that the signal noise as a percentage was significantly lower in the region of ±0.1%. However, over the 18–19-m lengths tested, this corresponds to a spatial uncertainty of around ±18–19 mm. If this is correct, then the helical implementation of the Er fibre presented here achieves a ten times higher spatial resolution, albeit over a considerably shorter measurement range.

## 6. Conclusions

An alternative method for measuring detonation velocity has been demonstrated, which uses easily obtainable Er-doped optical fibres as measurement probes. This method is simple to implement, cheap, scalable, minimally invasive, and relatively insensitive to strain.

One of the crucial factors when implementing this technique is the calibration, which should be undertaken in order to determine the optimal pump characteristics and dopant concentration for a given measurement length and for a maximum linearity. In these experiments, the calibration was achieved using laboratory cutback tests at a variety of pump powers.

The results of these cutback tests on high-concentration Er/Yb fibre (1000 dBm/m absorption at 980 nm) demonstrate the potential for these probes to be used as high spatial resolution velocity sensors, with a response that is linear to within approximately ±20 μm. The practical difficulty, in this case, is to pickup or amplify the low levels of total fluorescence.

Once an initial calibration has been done, the active fibre probe is easy to implement and analyse. In this work, a 1-m length of Er-doped fibre was used to measure the detonation velocity to within 2% of the expected value and with a maximum spatial uncertainty of around ±2 mm.

## Figures and Tables

**Table 1:** A comparison of the optimal parameters (pump power and length) for three fibres with different pump absorption: Er Fibrecore I-12 (11.6 dB/m), Er Fibrecore I-25 (23.9 dB/m), and in-house fabricated Er/Yb (>1000 dB/m).

Absorption	Pump Power	Length	Initial ASE Power
dB/m (at 980 nm)	mW	cm	(Approx.)
11.6	2.9–3.7	100	−25 dBm
23.9	2.5–3.3	10	−32 dBm
>1000	14.1–16.6	1	−40 dBm

## Abbreviations

The following abbreviations are used in this manuscript:

ASE	Amplified Spontaneous Emission
CFBG	Chirped Fibre Bragg Grating
DAQ	Data Acquisition Device
TOA	Time of Arrival
WDM	Wavelength Division Multiplexing

## References

[1] Handley C.A., Lambourn B.D., Whitworth N.J., James H.R., Belfield W.J. (2018). Understanding the shock and detonation response of high explosives at the continuum and meso scales. Appl. Phys. Rev..

[2] Suceska M. (1997). Experimental determination of detonation velocity. Fragblast.

[3] Shinas M.A., Doty D.L. (2018). 1550nm fiber optic TOAD (time of arrival diagnostic) for measuring sub-nanosecond resolution of detonation break out. AIP Conf. Proc..

[4] Benterou J., Udd E., Wilkins P., Roeske F., Roos E., Jackson D. (2007). In-Situ Continuous Detonation Velocity Measurements Using Fiber-Optic Bragg Grating Sensors.

[5] Gilbertson S., Jackson S.I., Vincent S.W., Rodriguez G. (2015). Detection of high explosive detonation across material interfaces with chirped fiber Bragg gratings. Appl. Opt..

[6] Magne S., Lefrancois A., Luc J., Laffont G., Ferdinand P. Real-time, distributed measurement of detonation velocities inside high explosives with the help of chirped fiber Bragg gratings. Proceedings of the Fifth European Workshop on Optical Fibre Sensors.

[7] Wei P., Lang H., Liu T., Xia D. (2017). Detonation velocity measurement with chirped fiber Bragg grating. Sensors.

[8] Rodriguez G., Gilbertson S. (2017). Ultrafast fiber Bragg grating interrogation for sensing in detonation and shock wave experiments. Sensors.

[9] Weiss J.D. (1994). Impurity-doped fiber-optic shock position sensor. J. Lightw. Technol..

[10] Fan N., Huang S., Alavie A. (1995). Rare earth doped fibre for structural damage assessment. Smart Mater. Struct..

[11] Sargis P., Molau N., Sweider D., Lowry M., Strand O. (1999). Photonic Doppler Velocimetry.

[12] Frugier P.A., Mercier P., Bénier J., Veaux J., Debruyne M., Rion C., Dubreuil E. PDV and shock physics: Application to nitro methane shock-detonation transition and particles ejection. Proceedings of the SPIE Optical Engineering + Applications.

